# Benchmarking accelerated next-generation sequencing analysis pipelines

**DOI:** 10.1093/bioadv/vbaf085

**Published:** 2025-05-15

**Authors:** Pubudu Saneth Samarakoon, Ghislain Fournous, Lars T Hansen, Ashen Wijesiri, Sen Zhao, Rodriguez Alex A, Tarak Nath Nandi, Ravi Madduri, Alexander D Rowe, Gard Thomassen, Eivind Hovig, Sabry Razick

**Affiliations:** Scientific Computing Services, Division for Research, Dissemination and Education, University of Oslo, Oslo, 0373, Norway; Norwegian National Unit for Newborn Screening, Division for Pediatric and Adolescent Medicine, Oslo University Hospital, Oslo, 0450, Norway; Scientific Computing Services, Division for Research, Dissemination and Education, University of Oslo, Oslo, 0373, Norway; Scientific Computing Services, Division for Research, Dissemination and Education, University of Oslo, Oslo, 0373, Norway; Department of Tumor Biology, Institute for Cancer Research, Oslo University Hospital, Oslo, 0424, Norway; Data Science and Learning, Argonne National Laboratory, University of Chicago, Chicago, IL, 60439, United States; Data Science and Learning, Argonne National Laboratory, University of Chicago, Chicago, IL, 60439, United States; Data Science and Learning, Argonne National Laboratory, University of Chicago, Chicago, IL, 60439, United States; Norwegian National Unit for Newborn Screening, Division for Pediatric and Adolescent Medicine, Oslo University Hospital, Oslo, 0450, Norway; Division for Research, Dissemination and Education, University of Oslo, Oslo, 0373, Norway; Department of Tumor Biology, Institute for Cancer Research, Oslo University Hospital, Oslo, 0424, Norway; Centre for Computational Inference in Evolutionary Life Science (CELS), Department of Informatics (USIT), University of Oslo, Oslo, 0373, Norway; Scientific Computing Services, Division for Research, Dissemination and Education, University of Oslo, Oslo, 0373, Norway

## Abstract

**Motivation:**

Industry-standard central processing unit (CPU)-based next-generation sequencing (NGS) analysis tools have led to longer runtimes, affecting their utility in time-sensitive clinical practices and population-scale research studies. To address this, researchers have developed accelerated NGS platforms like DRAGEN and Parabricks, which have significantly reduced runtimes—from days to hours. However, these studies have evaluated accelerated platforms independently without sufficiently assessing computational resource usage or thoroughly investigating speedup scalability, a gap our study is designed to address.

**Results:**

Corroborating previous studies, accelerated pipelines demonstrated shorter runtimes than CPU-only approaches, with Parabricks-H100 demonstrating the highest speedups, followed by DRAGEN. In mapping, DRAGEN outperformed Parabricks (L4 and A100) and matched H100 speedups. Parabricks (A100 and H100) variant calling demonstrated higher speedups than DRAGEN. Moreover, DRAGEN and Parabricks-H100 mapping showed positive trends in the coverage-based scalability analysis, while other configurations failed to scale effectively. Our profiler analysis provided new insights into the relationships between Parabricks’ performances and resource usage patterns, revealing its potential for further improvements. Our findings and cost comparison help researchers select accelerated platforms based on coverage needs, timeframes, and budget, while suggesting optimization strategies.

**Availability and implementation:**

Datasets are described in the ‘Data availability’ section. Our NGS pipelines are available at https://github.com/NAICNO/accelerated_genomics.

## 1 Introduction

Next-generation sequencing (NGS) has emerged as a revolutionary technology for determining the order of nucleotides in a genome at an unprecedented rate. Recent technological advancements in this field have generated massive amounts of highly accurate NGS datasets, significantly impacting current clinical and research practices. Currently, NGS analysis pipelines are routinely used in clinical settings, enabling the comprehensive identification of disease-causing variants in patients. It also drives population-scale research studies that improve our understanding of human diseases.

Industry-standard, best-practice NGS analysis pipelines employ numerous bioinformatics tools (Krusche *et al.* 2019, Koboldt 2020). Typically, these tools have been developed to utilize central processing units (CPUs) for computational power (McKenna *et al.* 2010, Li 2013). Owing to the physical limitations of CPU platforms, the turnaround time for analysing a single sample via the CPU-only Genome Analysis Toolkit (GATK) best-practice pipeline can be up to several days (Bourchany *et al.* 2017, Kumaran *et al.* 2019). This turnaround time presents a substantial challenge, creating a bottleneck in the current clinical genomic and research practices. However, in recent years, several bioinformatics tools have been developed to leverage the power of field-programmable gate arrays (FPGAs) and graphical processing units (GPUs) for computing tasks and to reduce the runtime of NGS analysis (Franke and Crowgey 2020, Zhao *et al.* 2020, Alganmi and Abusamra 2023). For example, Illumina DRAGEN (Dynamic Read Analysis for GENomics) (Illumina, Inc. n.d.), NVIDIA Clara™ Parabricks (hereafter Parabricks) (NVIDIA Corporation 2024a), Sentieon (Freed *et al.* 2017), and BaseNumber (Zhang *et al.* 2021) are such tools developed to overcome the turnaround time bottleneck.

DRAGEN is a combination of a highly configurable FPGA hardware platform and a software suite that accelerates the performance of genomics analysis. Several studies have demonstrated a significant improvement in the runtime performance of DRAGEN compared to CPU-based pipelines. For instance, recent benchmark studies have shown speedups of 17–33 for DRAGEN relative to CPU-native pipelines (Zhao *et al.* 2020, Betschart *et al.* 2022). In terms of variant calling efficiency, DRAGEN has shown comparable or superior performance to CPU-native GATK (https://gatk.broadinstitute.org/)-based pipelines across multiple studies. For example, F1-score (harmonic mean of precision and recall [Christen *et al.* 2024]), ranging from 0.985 to 0.992 for single nucleotide variant (SNV) calling in DRAGEN, was comparable to that of other methods (Zhao *et al.* 2020). Another study showed higher F1 scores for DRAGEN SNV calling compared to the CPU-based GATK pipeline, especially in difficult-to-map regions (Betschart *et al.* 2022). These studies collectively highlight DRAGEN’s importance in accelerating NGS pipelines while maintaining high accuracy in variant calling.

In the realm of GPU acceleration, Parabricks has become one of the most commonly used software suites in NGS pipelines (Tanjo *et al.* 2021, Carpi *et al.* 2022, O’Connell *et al.* 2023). The massively parallel processing capabilities of the GPUs coupled with the Parabricks software suite have enabled rapid processing of large-scale genomics datasets. Therefore, Parabricks-based pipelines have demonstrated a significant reduction in turnaround time of NGS analysis. For instance, one of the early comparative analyses showed a 35-fold speedup for the Parabricks pipeline compared to a CPU-only pipeline (Franke and Crowgey 2020). In addition, another benchmark study agreed with earlier findings showing 21-fold speedup in Parabricks variant calling compared to the CPU-only GATK pipeline (Rosati 2020). A more recent publication demonstrated a 65-fold speedup for Parabricks relative to CPU-only pipelines (O’Connell *et al.* 2023). When considering the variant calling accuracy, a performance evaluation of Parabricks using NA12878 Platinum Genomes ground truth showed 99.8% SNV calling accuracy and 99.1% small insertion/deletion (indel) calling accuracy (O’Connell *et al.* 2023). Moreover, the Parabricks-based pipeline on Nanopore sequence datasets was consistent with the results of previous benchmarking studies, achieving 99.83% and 92.11% precision and 99.66% and 60.77% sensitivity for SNVs and indels, respectively (Goenka *et al.* 2022). These studies highlight the importance of Parabricks in significantly reducing the runtime of NGS pipelines without compromising variant calling accuracy.

According to recent publications (Franke and Crowgey 2020, Rosati 2020, Zhao *et al.* 2020, Tanjo *et al.* 2021, Betschart *et al.* 2022, Carpi *et al.* 2022, Goenka *et al.* 2022, O’Connell *et al.* 2023), both DRAGEN and Parabricks can be positioned as powerful tools for large-scale genomic studies and time-sensitive clinical applications. However, these studies have evaluated the NGS acceleration capabilities of these two platforms separately. Therefore, there is a pressing need for a comprehensive, comparative analysis of these two platforms alongside traditional CPU-based pipelines. We aimed to address this gap by presenting a thorough benchmark of CPU-only, Parabricks, and DRAGEN pipelines in the context of whole-genome sequencing (WGS)-based SNV and indel calling. Here, we comprehensively assess DRAGEN and Parabricks, the two most widely used platforms in research and clinical settings, without providing an exhaustive review of all commercial accelerated genomics platforms. Our evaluation focuses on multiple factors, including runtime performance and speedup of accelerated pipelines relative to CPU-only baseline, variant calling accuracy, and scalability across high- and low-coverage datasets. Furthermore, we provide an in-depth analysis of the resource usage specific to Parabricks, offering valuable insights into GPU and other system requirements. This comprehensive assessment of accelerated NGS pipelines, encompassing a range of critical aspects, helps guide researchers and clinicians when implementing accelerator technologies, ultimately contributing to more efficient genomic studies and diagnostic workflows.

## 2 Methods

### 2.1 Benchmark dataset

We evaluated the accelerated NGS pipelines against the CPU-only pipeline using raw sequence data (FASTQ) of 10 WGS samples. These datasets were sourced from two prominent genomic projects: Illumina Platinum Genomes (high-coverage WGS) (Eberle *et al.* 2017) and the 1000 Genomes Project Phase 3 (low-coverage WGS). We accessed six samples from Illumina Platinum Genomes high-coverage WGS datasets and four low-coverage WGS samples from the 1000 Genomes Project Phase 3 release (more information is detailed in Supplementary Table S1).

### 2.2 CPU-only pipeline

**Figure 1.**
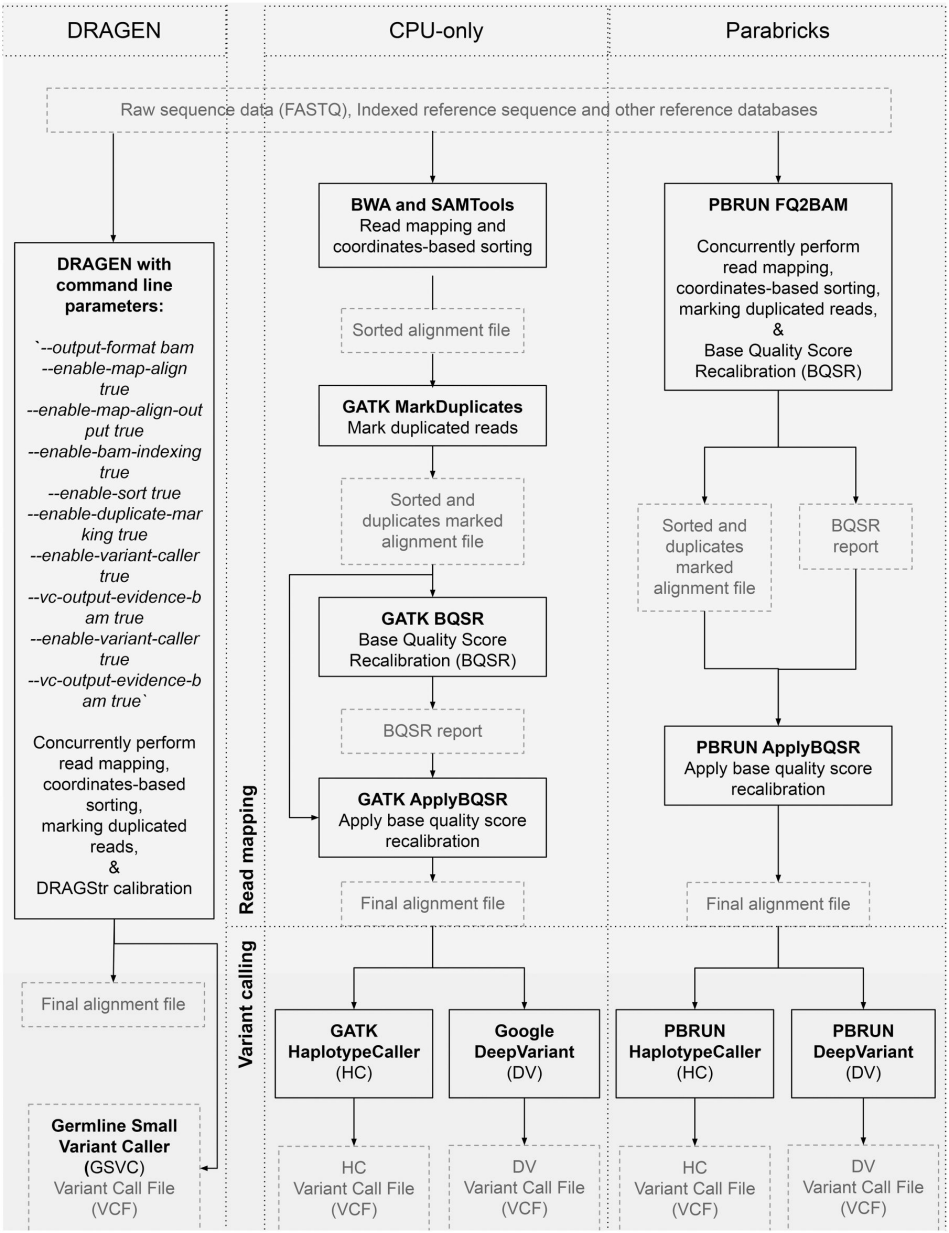
Overview of the three NGS pipelines—DRAGEN, CPU-only, and Parabricks. The workflow diagram compares three NGS pipelines, showing the input and output at each processing step.

We implemented a gold-standard CPU-based germline variant calling pipeline following best-practice recommendations (Van der Auwera *et al.* 2013, Krusche *et al.* 2019, Koboldt 2020) (Fig. 1). The command line parameters of our CPU-only pipeline were set according to one of the most commonly used best-practice workflow-nf-core/sarek (Garcia *et al.* 2020). The pipeline accepts raw sequencing data (FASTQ) as the main input. Initial read alignment was performed by mapping raw paired-end reads to GRCh38 Primary Assembly reference sequence using BWA (Burrows-Wheeler Aligner) (Li and Durbin 2009), followed by processing with SAMtools (Li *et al.* 2009) to convert the output to BAM format and sort the aligned reads (Li and Durbin 2009). The sorted alignment file was then processed through a series of downstream steps using the GATK (release 4.0.3.0; McKenna *et al.* 2010, Van der Auwera *et al.* 2013, Zhao *et al.* 2020). Here, GATK MarkDuplicates was first applied to identify and flag duplicate reads. Next, we performed base quality score recalibration (BQSR) using GATK BaseRecalibrator to generate a recalibration report, which was then applied to the alignment file using GATK ApplyBQSR. This process resulted in a final BAM file with duplicates marked and base quality scores recalibrated. Finally, we identified SNVs and indels using two variant callers: GATK HaplotypeCaller (HC; DePristo *et al.* 2011) and DeepVariant (DV; Poplin *et al.* 2018).

### 2.3 Parabricks pipeline

We implemented a GPU-accelerated germline variant calling pipeline leveraging the NVIDIA Parabricks platform (NVIDIA Corporation 2024a). To maintain compatibility across pipelines, our Parabricks (version 4.3.0-1) pipeline followed both best practices (Van der Auwera *et al.* 2013, Koboldt 2020) and vendor-specific guidelines (NVIDIA Corporation 2024a). Specifically, Parabricks FQ2BAM was implemented to concurrently perform multiple steps, including read mapping, duplicate marking and sorting, and generating BQSR report. Subsequently, BQSR report was used to apply the recalibration on the initial alignment file and to generate the final alignment file. Finally, Parabricks HC and Parabricks DV were implemented to call SNVs and indels (Fig. 1).

### 2.4 DRAGEN pipeline

We implemented the DRAGEN pipeline using a set of commands provided by the vendor (Illumina, Inc. n.d.) (Fig. 1). DRAGEN concurrently performs numerous processes compatible with the other two pipelines: load input FASTQ files and the reference, perform read mapping, and run post-alignment tasks including duplicate marking and sorting. However, DRAGEN differed from the other two pipelines in the ‘DRAGStr calibration’ step, which was implemented to model the indel error more accurately (Alganmi and Abusamra 2023). DRAGEN SNV and indel calling was performed by using the Germline Small Variant Caller (GSVC) (Illumina, Inc. 2017a).

The DRAGEN pipeline generates ‘Duration Metrics’ (Illumina, Inc. 2024a), reporting runtimes of each process. Due to the concurrent execution of read mapping-related processes, their aggregated runtime did not reflect the real time taken to complete the mapping stage. Therefore, to calculate the runtime of the DRAGEN read mapping and alignment refinement stage, we obtained real-time logs for three processes (each dependent on the output of its predecessor): loading reference datasets (Illumina, Inc. 2024b), performing read mapping (Illumina, Inc. 2024c), and generating alignment files (Illumina, Inc. 2024d). Conveniently, DRAGEN ‘Duration Metrics’ (Illumina, Inc. 2024a) directly provided the runtime of the variant calling stage.

### 2.5 Hardware configuration

The CPU-only pipeline was run on a server with AMD EPYC 7702 processors (Supplementary Table S2). For resource allocation, we initially followed the specifications recommended by nf-core/sarek (https://github.com/nf-core/sarek), a widely adopted bioinformatics workflow. Recent studies have shown that CPU-native NGS tools, despite supporting multi-threaded programming models, often exhibit limited scalability on modern multi-core HPC systems (Lee *et al.* 2016). Therefore, if a process failed due to exceeding the wall-time, CPU-core, or resident memory limit, we adjusted resource allocations in subsequent runs based on the observed requirements.

The Parabricks pipeline was run on four NVIDIA L4 (Ada Lovelace architecture—24 GB), four A100 GPUs (Ampere architecture—40 GB GPUs), and eight H100 (Hopper architecture—80 GB) GPUs. L4 GPUs were accessed via a G2 Google Compute Engine machine on Google Cloud Platform (GCP), A100 GPUs were available on an HPC cluster with AMD EPYC 7702, 64-Core processors (UiO HPC), and H100 GPUs were accessed via a GCP A3 Google Compute Engine machine.

The DRAGEN pipeline was run on an Illumina DRAGEN server V2 with Dual Intel Xeon Gold 6126 for a total of 48 threads, equipped with a total of 256 GB RAM and running with the Centos V7 operating system and the DRAGEN software v4.2. Data used for the processing were stored on an internal 2TB Intel NVME P4600 disk.

### 2.6 Speedup factor calculation

We measured the real-time (wall-clock time) taken for different stages of our NGS pipelines. The speedup factor is then calculated by dividing the real time of the baseline CPU process by the real time of accelerated processes, providing a clear assessment of the performance improvements achieved through hardware acceleration.

### 2.7 Variant calling performance evaluation and concordance analysis

We used hap.py (https://github.com/Illumina/hap.py; v0.3.15) with the ‘vcfeval comparison engine’ to compare diploid genotypes at the haplotype level and generated performance metrics for both performance evaluation and concordance analyses. Hap.py accepts two input VCF files—a query and a ground-truth VCF file—to identify true positives (TP), false positives (FP), and false negatives (FN). These classifications are determined based on variant matching stringencies, specifically the ‘genotype match’ criteria, as defined within the tool (Krusche 2017). The precision, recall and F1-score reported in the performance metrics were also calculated within the Hap.py tools as TP/(TP + FP), TP/(TP + FN) and 2 × TP/(2 × TP + FN + FP), respectively.

During the performance evaluation, the gold-standard truth call-sets (high-confidence variants) and high-confidence genomic interval files of NA12877 and NA12878 WGS samples were obtained from the Illumina ‘Platinum Genomes’ (Eberle *et al.* 2017) project’s Amazon Web Services S3 bucket (Illumina, Inc. 2017b). The high-confidence genomic interval files were used to limit the search space of hap.py runs in both analyses.

The performance evaluation helped determine the validity of the results from our NGS pipelines by comparing them against gold-standard truth call-sets. Here, we compared VCF results files from all three NGS pipelines against the Illumina Platinum high-confidence variants. In other words, we used VCF outputs of our NGS pipelines as query files and Illumina platinum high-confidence VCF files as the ground truth of the hap.py tool. During the concordance analysis, we evaluated the consistency between accelerated variant callers and best-practice tools by comparing VCF results files from accelerated pipelines against those from the CPU-only pipeline (i.e. VCF files of accelerated pipelines as query and the CPU-only best-practice output VCF files as the ground truth of the hap.py tool).

### 2.8 System resource profiling

We implemented Jobanalyzer (https://github.com/NAICNO/Jobanalyzer)—an easy-to-use resource usage reporting tool—as the profiler in our study. Additionally, it offered a comprehensive interface for post-hoc job analyses supporting profile visualization.

Jobanalyzer probes the system at predefined intervals (series of time points) and gathers statistics on resource usage. We chose 30-s intervals to profile processes on the GCP VMs with L4 and H100 GPUs. Our computing node with A100 GPUs uses 5-min intervals for long-term cluster monitoring. To ensure consistency of profiler configurations and minimize system variability, we confined our GPU resource usage and coverage-based scalability analysis to L4 and H100 profiles, excluding A100 profiles.

Jobanalyzer’s monitoring approach poses the risk of not recording system usage fluctuations that may occur within the probing intervals. To examine these blind spots, we submitted five samples on L4 GPUs in a Google VM (L4-VM) implementing Jobanalyzer with 3-s intervals (high-frequency profiler). This extended analysis, as detailed in Supplementary Fig. S1, corroborated our initial profiling findings, reinforcing the insights presented in the Results and Discussion sections.

In contrast to Parabricks, the vendor-locked nature of the DRAGEN hardware platform prevented us from running a profiler alongside the DRAGEN runs. Therefore, our resource profile analysis was limited to the Parabricks pipelines.

## 3 Results

We systematically compared three NGS pipelines (CPU-only, Parabricks, and DRAGEN) using 10 WGS samples (detailed in the Methods section). While these pipelines were designed following best-practice recommendations, implementation of their individual steps differed due to vendor-specific guidelines (detailed in the methods). Therefore, to use a common framework for our comparative evaluation, we consolidated the pipeline steps into two main stages—read mapping and alignment refinement (hereafter ‘read mapping’) and variant calling (Fig. 1).

### 3.1 Runtime performance analysis

**Figure 2.**
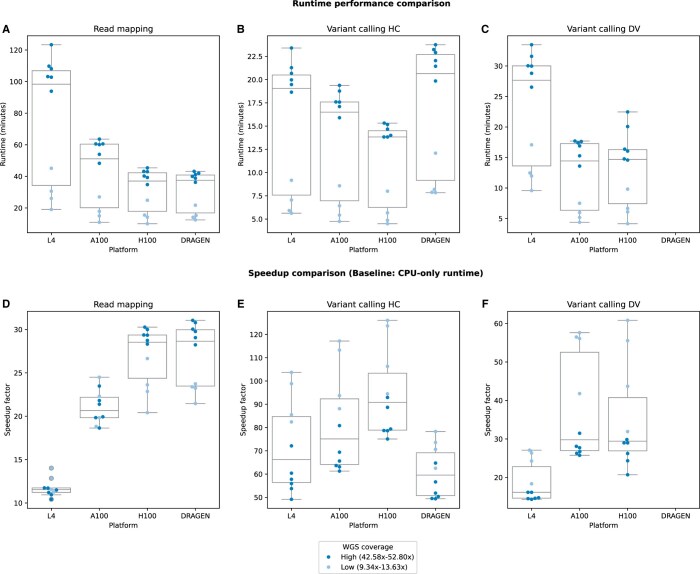
Runtime and speedup comparison. (A–C) Runtime comparisons (in minutes) across accelerated platforms for (A) read mapping stage, (B) variant calling HC, and (C) variant calling DV processes. Accelerated platforms include Parabricks on L4, A100, and H100 GPUs and DRAGEN. CPU-only pipeline data omitted to highlight runtime differences between accelerated pipelines. (D–F) Speedup factors of accelerated (D) read mapping stage, (E) variant calling HC and (F) variant calling DV, calculated relative to CPU-only baseline (Speedup = baseline runtime/accelerated runtime).

The CPU-only pipeline, which served as our baseline, consistently exhibited the longest runtimes in read mapping and variant calling stages. As expected, both accelerated pipelines (DRAGEN and Parabricks) exhibited significantly lower runtimes across all samples (Supplementary Table S3). When comparing two accelerated mapping processes, DRAGEN outperformed Parabricks on L4 and A100 but showed similar runtimes to H100 (Fig. 2A). In variant calling, Parabricks HC (on all three GPU platforms) showed lower runtimes than DRAGEN GSVC (Fig. 2B). Parabricks mapping and HC performances across different GPU platforms were consistent with expectations, where L4 had the highest runtimes, followed by A100, with H100 achieving the lowest runtimes (Fig. 2A and B). In contrast, Parabricks DV on A100 runtimes were lower than those on H100 in most samples (Fig. 2C).

We calculated the total runtime by aggregating the processing time of the read mapping and haplotype-based variant calling stages (Supplementary Table S3C). In other words, Parabricks DV was excluded in the total runtime calculation as a compatible variant caller was not implemented in the DRAGEN pipeline. The shortest total runtimes were reported for Parabricks pipeline on H100, followed by DRAGEN. As anticipated, the total runtime of Parabricks pipelines decreased with improving GPU technology.

### 3.2 Speedup factor analysis

As detailed in the previous section, both accelerated pipelines demonstrated significantly reduced runtimes relative to the CPU-only baseline. In Parabricks read mapping, substantially reduced runtimes on L4, A100, and H100 GPUs translate to speedups of ∼10–14, ∼19–24, and ∼20–30, respectively (Fig. 2D). For DRAGEN read mapping, the observed speedup ranged from ∼23 to 31 (Fig. 2D). Accelerated variant callers showed higher speedup factors than accelerated read mapping (Fig. 2E and F). For instance, DRAGEN GSVC exhibited ∼49–78 speedup, and Parabricks HC on L4 and A100 GPUs displayed ∼49–117 speedup factors. Parabricks HC on H100 GPUs exhibited the most remarkable speedup (∼75–126) among all tested configurations (Fig. 2E). In contrast, Parabricks DV diverged from the expected pattern in most samples (Fig. 2F) by showing higher speedups on A100 (∼26–58) than on H100 GPUs (∼21–61).

### 3.3 Coverage-based scalability of accelerated pipelines

The depth of coverage is one of the key factors affecting the processing time of NGS analysis (Sims *et al.* 2014). Therefore, we studied the scalability of our accelerated pipelines across low- and high-coverage WGS samples. As visualized in Fig. 2A–C, runtimes of high-coverage samples were higher than those of low-coverage samples, suggesting a direct relationship between input coverage and processing times. Analysis of speedup metrics (Fig. 2D–F) revealed three distinct performance patterns across platforms: (i) Parabricks read mapping speedup on L4 and A100 GPUs showed no clear relationship with high- and low-coverage samples; (ii) H100 and DRAGEN read mapping achieved superior speedup with high-coverage samples; and (iii) both Parabricks and DRAGEN demonstrated reduced speedup factors for variant calling in high-coverage samples. To comprehensively evaluate the speedup-coverage relationship, we examined how speedup varies with the average coverage of these 10 samples (Supplementary Fig. S2A–C). This analysis agreed with coverage-speedup patterns observed in Fig. 2D–F and revealed: (i) speedup of read mapping on H100 GPUs and DRAGEN platform scaled-up with average coverage; (ii) speedup and average coverage didn’t show a clear relationship for read mapping on L4 and A100 GPUs; (iii) Parabricks (on all GPUs) and DRAGEN variant calling speedup decreased with the increasing average coverage.

Next, we examined the relationships between speedup, number of identified variants, and the average coverage to determine the potential link among these factors. As shown in Supplementary Fig. S2D, speedup decreased with the increasing number of variants called in all three accelerated callers (a pattern similar to the speedup versus coverage behaviour). We also observed that the number of identified variants increased with the average coverage (Supplementary Fig. S2E). Since the variant calling processes depend on the sample’s coverage (detailed in the discussion), we could determine that the scalability of the speedup relies on the coverage, rather than the variant counts.

We assumed that the coverage-speedup relationship could be linked to the resource usage patterns of accelerated pipelines. Therefore, we designed the next stage of our analysis to gain more insight into the resource profiles of accelerated pipelines.

### 3.4 Coverage-dependent GPU resource usage in Parabricks

**Figure 3.**
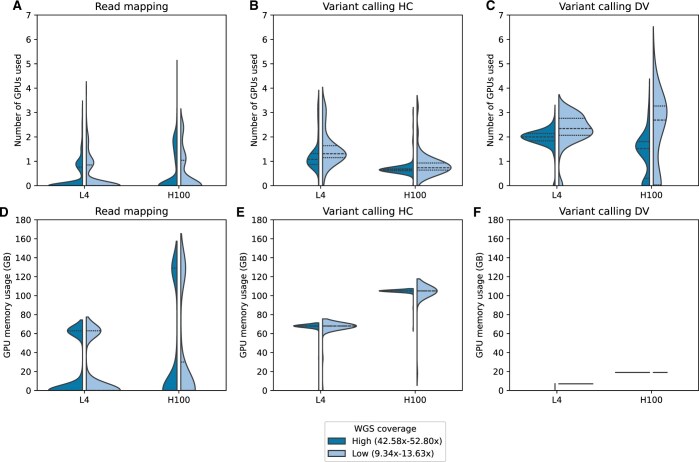
Resource usage patterns across coverage and GPU platforms. (A–C) L4 and H100 GPU (cards) usage comparison between low- and high-coverage samples during (A) read mapping stage, (B) variant calling HC, and (C) variant calling DV processes. (D–F) L4 and H100 GPU memory usage comparison for low- and high-coverage samples during (D) read mapping stage, variant calling (E) HC, and (F) variant calling DV processes.

To further study the speedup-coverage relationship in the context of computational resource usage, we performed coverage-based post-hoc job analysis using GPU and GPU memory profiles obtained from L4 and H100 platforms (detailed in the Methods section). Analysis of these resource profiles from high- and low-coverage samples revealed two GPU usage patterns: (i) high-coverage samples displayed high GPU usage during the read mapping stage on H100 GPUs (Fig. 3A) and (ii) low-coverage samples showed high GPU usage in Parabricks HC and DV (Fig. 3B and C). These two patterns align with our speedup-coverage analysis and may potentially explain Parabricks’ coverage-based scalability behaviour (detailed in the discussion).

Furthermore, GPU and GPU memory profiles revealed frequent measurements of zero or near-zero (Fig. 3A and D) in the read mapping stage, while variant calling stages maintained more consistent above-zero levels (Fig. 3B, C, E, and F). This observation was further supported by Supplementary Fig. S3, which illustrated more frequent periods of inactivity (zero usage) followed by spikes in GPU usage during the mapping process. Conversely, as shown in Supplementary Fig. S3, GPU usage during the variant calling stages was consistently over zero levels.

**Figure 4.**
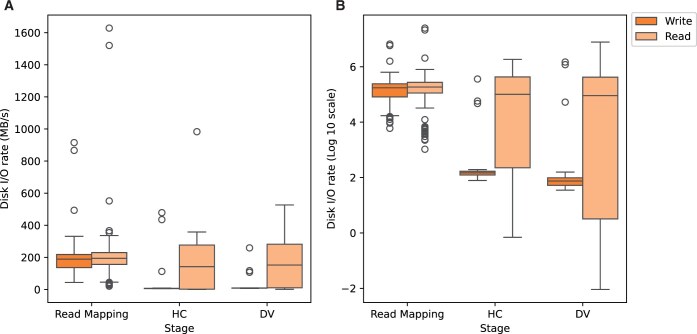
Disk I/O usage of Parabricks pipeline (on H100 GPUs). (A) Disk I/O rate distribution across three processes of the Parabricks pipeline; (B) same distribution in log10 scale to highlight read/write activity across the Parabricks pipeline.

The irregular GPU usage observed in the mapping process may result from disk I/O performance constraints due to its large-scale read–write operations. Therefore, we studied the data movement demands of the Parabricks pipeline by examining the disk I/O characteristics obtained from Google Compute instances with H100 GPUs (Fig. 4). The disk I/O performance of the Parabricks read mapping stage demonstrated high read and write activity, a common pattern observed in typical bioinformatics tools (Lee *et al.* 2016). Variant calling processes (Parabricks HC and DV) showed significantly higher ‘read’ than ‘write’ activity. To gain further insights, we visualized disk I/O characteristics along the runtime of Parabricks pipeline. As depicted in Supplementary Fig. S4, disk-read and write rates of Parabricks mapping process fluctuate significantly over time, while Parabricks HC and DV exhibited read–write asymmetry with read dominance. This may have implications on the work-load parallelization across GPUs (addressed in the Discussion section).

### 3.5 Performance evaluation and concordance analysis of variant callers

**Figure 5.**
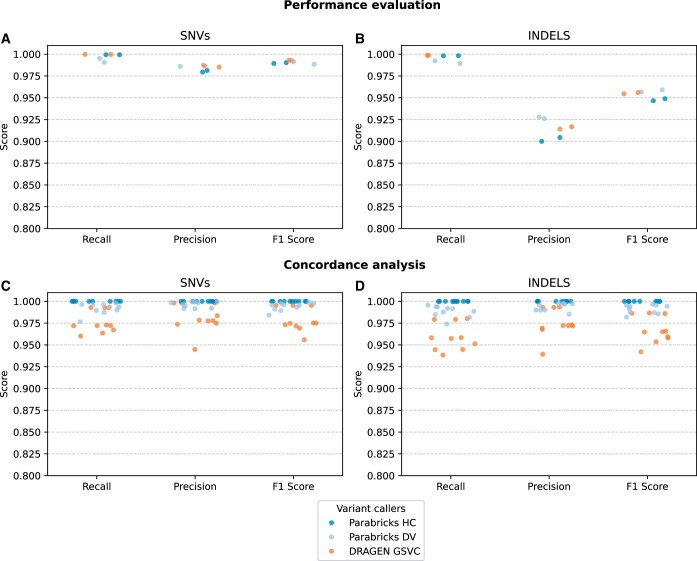
Performance evaluation and concordance analysis. (A, B) Precision, recall, and F1 scores from performance evaluation analysis of (A) SNVs and (B) indels. Performance evaluation analysis with the hap.py tool used VCF files of accelerated pipelines as query and the gold-standard high-confidence VCF files as the ground truth; (C, D) precision, recall, and F1 scores from concordance analysis of (C) SNVs and (D) indels. Concordance analysis with the hap.py tool used VCF files of accelerated pipelines as query and the CPU-only best-practice output VCF files as the ground truth.

We conducted the performance evaluation and concordance analysis to assess the validity and reliability of variant callers implemented in our NGS pipelines (detailed in the Methods section). The performance evaluation that used gold-standard ground truth (high-confidence variants) demonstrated near-perfect recall scores and slightly lower precision, leading to strong overall F1 scores for SNVs (Fig. 5). As expected, indel calling performance was lower than that of the SNVs. This is likely because the read alignment around indel is more divergent, making precise calling a relatively challenging task (Fang *et al.* 2014, Li 2014). According to the SNV and indel calling concordance analysis that compared accelerated callers to CPU-only callers (Fig. 5), Parabricks HC achieved near-perfect scores across all metrics. Parabricks DV also displayed strong concordance, with high scores. Although DRAGEN GSVC showed slightly lower concordance than other callers, it still maintained a high accuracy (F1 scores >0.93). As further detailed in the Discussion section, both performance evaluation and concordance analysis help determine the validity and consistency of the results from pipelines tested in this study.

## 4 Discussion

We comprehensively evaluated accelerated (DRAGEN and Parabricks) NGS pipelines, revealing their notable similarities and significant differences. As highlighted in the Results section, we focused our study on several key performance indicators: runtime and speedup, coverage-based scalability and system resource usage, and variant calling performance.

In this study, we implemented DRAGEN on vendor-specific hardware and Parabricks across disparate GPU infrastructures, evaluating their real-world use cases. Both accelerated pipelines exhibited significant speedup factors relative to the CPU-only baseline and agreed with previous studies (Franke and Crowgey 2020, Rosati 2020, Zhao *et al.* 2020, Betschart *et al.* 2022). These findings underscored the importance of accelerated pipelines in improving the runtime performance of large genomics studies. Furthermore, these results have implications in clinical genomics settings, as they demonstrate turnaround time reductions with speedups up to 126 (Fig. 2). This is crucial for time-sensitive prognosis and addresses one of the main concerns in WGS-based genetic diagnostics (Saunders *et al.* 2012, Riess *et al.* 2024).

Although Parabricks and DRAGEN pipelines offer significant performance boosts, our analysis revealed notable differences in speedup between the two pipelines. Specifically, DRAGEN read mapping outperformed Parabricks on L4 and A100 GPUs. This superior performance can likely be attributed to its custom mapping algorithm, which is optimized for dynamic seed extension and iterative querying (Illumina, Inc. 2023a, 2023b). Compared to the BWA algorithm (Li and Durbin 2009), a customized dynamic seed extension reduces redundant mapping efforts (Behera *et al.* 2024), while iterative querying with extended seeds leads to dramatically fewer index memory accesses (Miller *et al.* 2015). Both of these algorithmic optimizations may contribute to shorter DRAGEN mapping runtimes compared to Parabricks’ GPU-optimized BWA algorithm (NVIDIA Corporation 2024b) on L4 and A100 GPUs. However, the advancements in GPU technology seem to have bridged the performance gap in accelerated read mapping processes, as DRAGEN and Parabricks-H100 exhibited similar speedups for this stage. In contrast to read mapping, the variant calling speedup of Parabricks on A100 and H100 GPUs surpassed that of DRAGEN. Therefore, the two pipelines demonstrated unique strengths in different aspects of genomic data processing.

Accelerated variant callers tested in our study exhibited decreasing speedup as variant counts increased (Supplementary Fig. S2D). Additionally, variant counts identified by these callers increased proportionally with the average coverage (Supplementary Fig. S2E). These observations corroborated previous research on the coverage–variant count relationship (Ajay *et al.* 2011). As demonstrated in earlier work, higher average coverage increases the percentage of ‘callable genomic loci’ (regions with sufficient coverage), consequently yielding higher variant counts (Ajay *et al.* 2011). Thus, the speedup of accelerated variant callers depended more on average coverage than on variant counts. As discussed later in this section, we further examined coverage–speedup relationship in the context of GPU usage and disk I/O profiles.

Speedup factor comparison across different pipeline stages (Fig. 2D–F) demonstrated higher speedup for variant calling processes than the read mapping stages. For instance, Parabricks HC on H100 GPUs reported ∼75–126 speedup, while the speedup of the mapping stage on the same GPUs ranged between ∼20–30. As shown in Supplementary Fig. S3, the read mapping stage exhibited frequent fluctuations between zero and above-zero GPU usage across runtime. In contrast, Parabricks HC consistently maintained above-zero GPU activity. Therefore, higher GPU usage of Parabricks HC (∼1–4 and ∼0.5–2 GPU cards on L4 and H100) may have contributed to its higher speedup relative to the read mapping stage that showed frequent GPU inactive and active periods.

Disk I/O profile of the Parabricks pipeline showed higher read/write activity in the mapping stage than that of HC and DV (Supplementary Fig. S4). It is plausible that frequent read/write operations during the mapping stage likely caused frequent periods of GPU idle time (i.e. GPUs becoming inactive while waiting for data). While this disk I/O activity coincides with GPU profiles, further analysis is needed to verify this relationship. However, due to Parabricks’ closed-source nature, we could not implement an application-level profiler or test an alternative data-feeding approach (i.e. making Parabricks read data directly from RAM). Consequently, we were unable to perform an in-depth investigation into the overall relation between speedup, GPU usage, and disk I/O profiles.

Our coverage-based scalability analysis revealed a clear relationship between speedup, average coverage, and GPU usage, especially in Parabricks read mapping on H100 GPUs. For instance, read mapping of high-coverage samples on H100 displayed high GPU usage (Fig. 3A) and high speedup (Supplementary Fig. S2A—H100 mapping speedup increased with the coverage). This pattern suggested that Parabricks effectively scales read mapping speedup on H100 potentially through efficient workload distribution across GPUs.

In contrast to the read mapping stage, accelerated variant callers showed the opposite patterns between coverage, speedup and GPU usage. For example, Parabricks HC and DV on high-coverage samples showed low GPU usage (Fig. 3B and C) and low speedups (Fig. 2E and F). These observations suggested that the Parabricks variant callers demonstrate limited speedup scalability with average coverage and GPU usage.

To investigate the scaling limitation of the variant calling stages, we implemented Parabricks HC with the ‘—run-partition’ parameter that splits the genome into multiple partitions for simultaneous processing. Since this partitioning method was not clearly described in the Parabricks documentation, we developed a proof-of-concept NextFlow workflow (NF-optimized workflow) that splits the genome by chromosome and runs Parabricks HC concurrently across multiple chromosomes. We evaluated these two approaches using high-coverage samples and compared their performance to the original implementation. As detailed in Supplementary Text 01. Fig. S1, both Parabricks HC with ‘—run-partition’ and our NF-optimized workflow showed higher GPU usage (∼18%–42%) than the original Parabricks HC implementation (∼8%–15% GPU usage). Evidently, splitting the genomic dataset into groups for concurrent processing enabled Parabricks HC to use GPUs more effectively. Consequently, both ‘—run-partition’ runs and the NF-optimized workflow achieved faster variant calling runtimes relative to original runtimes (Supplementary Text 01. Table S1). Importantly, despite being tested on high-coverage samples, ‘—run-partition’ runs exhibited the highest speedups reported in our study (∼245–329), followed by NF-optimized workflow (∼179–232). These results indicated that genome splitting followed by parallel processing further improved the speedup even with high-coverage samples. Moreover, Parabricks HC ‘—run-partition’ runs displayed read-write asymmetry (Supplementary Text 01. Fig. S2), which was similar to our initial observations (Fig. 4). Therefore, these analyses underscored the importance of employing genome splitting and parallel processing to improve GPU usage efficiency and scale variant calling speedup, despite exhibiting disk I/O characteristics similar to the original implementations.

CPU-only DV that implements the TensorFlow machine learning framework (Poplin *et al.* 2018) for hardware-optimized deep learning analysis (Abadi *et al.* 2016) diverged from the expected speedup pattern when accelerated on different GPU platforms. For instance, Parabricks DV showed higher speedups on A100 than H100 for most samples. However, since the Parabricks source code is not publicly available, it was challenging to directly identify the algorithmic factors contributing to DV’s superior performance on A100 GPUs. According to Parabricks release notes, the DV version 4.3.1-1 was released with increased GPU usage and kernel optimization improvements (Supplementary Table S4). Therefore, we implemented 3 runs on H100 GPUs with Parabricks DV.v4.3.1-1 and compared the performances on different DV versions. As shown in Supplementary Table S4, DV.v4.3.1-1 did not show an improvement in the runtime performance relative to our initial implementation (i.e. both DV versions reported runtimes in a similar range). This suggested that the algorithmic optimizations in the updated Parabricks DV did not translate to faster runtimes on H100 GPUs, and the source for its unexpected performance (i.e. shorter runtimes on A100 than H100) remained unclear.

Parabricks DV on A100 GPUs were implemented on UiO HPC resources, while H100 runs were implemented on GCP A3 machines (detailed in methods). To investigate whether UiO HPC-specific factors contributed to Parabricks DV’s unexpected runtime on A100 GPUs, we examined the reproducibility of its runtime performance across different HPC systems. Here, we implemented the same workflow on the Polaris HPC system at the Argonne Leadership Computing Facility (Supplementary Text 02). Parabricks DV A100 implementations on both UiO and Polaris HPC resources reported runtimes in similar ranges (∼5–15 min). The total runtimes of the two implementations were also comparable (∼10–75 min). This underscored the runtime performance reproducibility of Parabricks DV workflow on A100 GPUs in different HPC systems.

Performance comparison of the Parabricks pipeline across different GPU platforms exhibited less pronounced speedup on high-end GPUs (A100 and H100) relative to L4 (Supplementary Fig. S5). As underlined in recent studies, high-end GPUs often experience bottlenecks in the data transfer process (i.e. it is increasingly challenging to feed data at sufficient rates as the GPU technology increases). This can lead to frequent idle time and reduced overall performance (Mohan *et al.* 2021, Murray *et al.* 2021). Therefore, the unexpected speedup of Parabricks across different GPU platforms could likely stem from the scaling discrepancy between the GPU compute capability and the disk I/O bandwidth. However, relative to L4 GPUs baseline, Parabricks HC with the ‘–run-partition’ option and NF-optimized implementations achieved ∼3–5 speedups on H100 GPUs (Supplementary Text 01. Table S2), which were substantially higher than those shown in Supplementary Fig. S5. Despite disk I/O limitations, these results provide a foundation for exploring strategies like genome splitting to gain high speedups (via GPU resource usage optimization), which would greatly benefit the genomics community.

Our performance evaluation (Fig. 5) revealed high scores across Parabricks and DRAGEN variant callers, highlighting their ability to correctly predict TPs while minimizing FPs and FNs. Studying these aspects is crucial, especially in clinical settings, as FPs and FNs can lead to false conclusions in genetic diagnosis. As evident in the results (Fig. 5), all accelerated callers demonstrated a strong ability to identify TP SNVs and indels (i.e. near-perfect SNV calling recall; indel calling recall >0.98) agreeing with previously published studies (Zhao *et al.* 2020, O’Connell *et al.* 2023). The tendency to predict FPs was higher for indel than SNVs (low precision in indel calling). This can negatively impact genetic diagnostics workflows. To minimize FP calls, current NGS workflows employ hard-threshold-based VCF-filtering approaches (Koboldt 2020, Pedersen *et al.* 2021). The DRAGEN pipeline has the option to generate filtered variant call-sets, while users can implement third-party bioinformatics tools (e.g. BCFtools [Danecek *et al.* 2021]) to apply hard filtering on Parabricks calls. The choice of hard filters can vary depending on the pipeline’s clinical application. Therefore, we did not validate filtered variant call-sets, as it is beyond the scope of this study.

Due to the closed-source nature, Parabricks and DRAGEN users do not have access to the source code and rely solely on vendor-provided documentation. This inability to examine the source code poses significant challenges in assessing the robustness of their underlying bioinformatics operational logic across different use cases or in testing whether their decision-making processes contain unintended biases. Our concordance analysis (Fig. 5) helped address these limitations and provided empirical evidence for the degree of agreement between accelerated and best-practice variant callers’ performances. Our analysis revealed that Parabricks HC could accurately replicate best-practice results (with near-perfect scores), highlighting the strong alignment between Parabricks HC’s underlying bioinformatics operational logic and industry-standard best practices. While Parabricks DV’s SNV calling approach also strongly overlapped with best practices, the indel calling approach slightly deviated from its baseline. According to the Parabricks DV documentation, CPU-only DV with the ‘—make_examples_extra_args’ command would generate results with high concordance. However, since we set our CPU-only baseline pipeline’s command line parameters according to one of the most commonly used best-practice workflows (detailed in methods), we did not alter our baseline pipeline to achieve high concordance scores for a selected accelerated caller. The bioinformatics operational logic of DRAGEN GSVC exhibited the highest deviation from the haplotype-based best-practice calling approach (CPU-only HC), as indicated by its lowest concordance scores. DRAGEN GSVC documentation outlines distinctive features, such as GSVC error handling, that differentiate its bioinformatics operational logic from the best-practice approach (Illumina, Inc. 2017a). These differences may explain its low concordance. Notably, the concordance analysis could miss edge cases not covered in our test datasets and fail to identify the root causes of discordant results.

The operational costs of these platforms also play a critical role when implementing accelerated technologies. To address this, we performed a straightforward cost comparison (Supplementary Text 03) focusing on GCP’s hourly rates, an HPC cluster with A100, and the DRAGEN’s server and licensing fees. The cost of GCP GPU instances varies significantly depending on the GPU model (four L4 and eight H100 GPUs cost $4.01 and $48.77 per hour, respectively). Therefore, the total cost for running the Parabricks pipeline on 10 samples in an L4 instance was $83.42, while the total cost on H100 was $448.01. In other words, Parabricks runs on H100 are approximately 5.4 times more expensive than those running on L4. In contrast to this substantial cost difference, Parabricks speedup on H100 relative to L4 ranged between 1.5- and 2.5-fold (Supplementary Figs 1E, 2E, and 3E), which further signifies the importance of optimizing it to fully utilize GPU resources. On the other hand, the cost of running Parabricks on A100 GPU in an HPC cluster depends on the price of the GPU card and the associated maintenance expenses. For the purpose of comparison, we estimated the hourly cost per A100 GPU on an HCP cluster to be approximately 0.13–0.71 USD (Supplementary Text 03). The DRAGEN platform operates on a cost model driven by licensing fees. Supplementary Text 03 also provides a cost comparison between DRAGEN and Parabricks on A100 GPUs in an HPC cluster to help gain more insight into the associated cost with running accelerated NGS pipelines. Therefore, the choice of accelerator technology requires careful consideration of multiple factors ranging from technical and biological objectives to the workload and operational expenses.

Our study was limited to two main variant types—SNVs and indels. However, the scope of DRAGEN and Parabricks platforms extends to a broad range of tools, including QC analysis, structural variant calling, and other post-processing stages. Supplementary Text 04 provides a comparison of tools available in Parabricks and DRAGEN systems (in the context of germline NGS pipelines), which are the focus of our ongoing activities in the accelerated genomics space.

We developed several NGS pipelines using the NextFlow workflow management system (Di Tommaso *et al.* 2017). The main pipelines include: (i) a Parabricks mapping and variant calling pipeline, (ii) a compatible CPU-only pipeline, and (iii) a proof-of-concept NF-optimized Parabricks HC pipeline. Additionally, we created a supplementary quality control pipeline for internal QC tasks. All these four pipelines, along with accompanying documentation, are publicly available under the MIT License at https://github.com/NAICNO/accelerated_genomics, enabling users to implement them on their preferred platforms.

In this study, we conducted a side-by-side comparison of DRAGEN and Parabricks pipelines, demonstrating their runtime performances and speedup relative to CPU-only best-practice baseline. Our variant calling performance and concordance analysis revealed the validity and consistency of our results. We also highlighted how Parabricks’ performance varies across different GPU platforms. While our results align with previous findings, our profiling analysis provided valuable insights into the performance of the accelerated pipelines that have not been adequately investigated in previous studies. For instance, our profiling analysis demonstrated how GPU usage of the Parabricks pipeline aligned with the observed speedup, while our disk I/O examination revealed plausible factors that could affect their performances. Additionally, our coverage-based scalability analysis showed how sample coverage can impact accelerated pipeline’s speedup. Despite these potential limitations, our detailed analysis of Parabricks HC highlighted effective strategies like genome splitting, which improved GPU usage and delivered high speedups even with high-coverage samples. These findings, coupled with our detailed cost comparison, can guide researchers in selecting accelerated platforms based on their coverage requirements, timelines, and budgets while implementing optimization strategies from our publicly released pipelines.

## 5 Conclusions

Our comprehensive analysis explored Parabricks and DRAGEN pipelines across a broad range of aspects and presented numerous core findings, focusing on several key performance indicators: runtime performance, speedup factors, coverage-based scalability, computational resource usage, disk I/O performance, and variant calling efficiency and concordance. Moreover, Parabricks HC (with the ‘—run-partition’ parameter) and our proof-of-concept NF-optimized workflow demonstrated the potential for further performance improvements through tool optimization. Our findings offer valuable insights to help make informed decisions when adopting or upgrading accelerated pipelines, providing potential solutions to address crucial time-sensitive challenges in clinical genomics and research settings.

## Supplementary Material

vbaf085_Supplementary_Data

## Data Availability

Sources for raw sequence datasets, Illumina Platinum high-confidence variant call files, and high-confidence genomic interval files used in this study are listed in the footnote of Supplementary Table S1. NGS pipelines developed in this study are available at the GitHub repository—https://github.com/NAICNO/accelerated_genomics.
