# Supplementary material for: Benchmarking accelerated next-generation sequencing analysis pipelines
Source: Bioinform Adv. 2025 May 15;5(1):vbaf085. doi: 10.1093/bioadv/vbaf085 (PMC12092081; doi:10.1093/bioadv/vbaf085)
Supplement: vbaf085_Supplementary_Data [file vbaf085_supplementary_data.zip › Publication-ready_Supplementary materials-20250404.pdf]

Supplementary materials:

Benchmarking accelerated Next Generation Sequence (NGS) analysis pipelines

Supplementary tables

Supplementary Table S1: Benchmark datasets used in the study

Sample ID	Average Coverage	Description	Population
NA12878	47.55	Illumina Platinum pedigree	CEPH
NA12891	47.09	Illumina Platinum pedigree	CEPH
NA12892	50.50	Illumina Platinum pedigree	CEPH
NA12877	49.88	Illumina Platinum pedigree	CEPH
NA12889	52.80	Illumina Platinum pedigree	CEPH
NA12890	42.58	Illumina Platinum pedigree	CEPH
NA12778	9.82	Low coverage WGS	CEPH
NA12812	9.35	Low coverage WGS	CEPH
NA12829	13.63	Low coverage WGS	CEPH
NA12843	10.14	Low coverage WGS	CEPH

- CEPH: Utah residents with Northern and Western European ancestry
- Raw sequence datasets are available via - https://www.internationalgenome.org/data-portal/sample
    - To access FASTQ files, follow `www.internationalgenome.org/data-portal/sample/<Sample ID>` and select `Illumina Platinum pedigree` or `1000 Genomes phase 3 release` accordingly.
- Illumina Platinum Genome related resources: https://www.illumina.com/platinumgenomes.html
- Illumina Platinum highly-confidence variant call files and highly-confidence genomic interval files for NA12877 and NA12878 - https://illumina.github.io/PlatinumGenomes/?prefix=2017-1.0/hg38/small_variants/
- Reference sequence used in the read mapping stage:
    - GRCh38 Primary Assembly reference sequence
        - “GCA_000001405.15_GRCh38_no_alt_analysis_set.fna.gz”
        - (https://ftp.ncbi.nlm.nih.gov/genomes/all/GCA/000/001/405/GCA_000001405.15_GRCh38/seqs_for_alignment_pipelines.ucsc_ids)

Supplementary Table S2: Hardware configuration summary

	CPU-only pipeline	Parabricks pipeline	DRAGEN pipeline
**CPU**	AMD EPYC 7702 (64 cores)*	AMD EPYC 7702	2 Intel Xeon Gold 6126 (12 cores each)
**GPU**	NA	4 NVIDIA L4 (Ada Lovelace architecture - 24GB)*<br><br>4 A100 GPUs (Ampere architecture - 40GB GPUs<br><br>8 H100 (Hopper architecture - 80GB)*	NA
**Memory (RAM)**	128 - 256 GB*	A100 GPUs: 412 GB<br><br>L4: 192 GB<br><br>H100: 1872 GB	256 GB
**GPU memory**	NA	A100 GPUs:<br><br>L4: 96 GB (GDDR6)<br><br>H100: 640 GB (HBM3)	NA
**Storage**	GPFS	GPFS	Local nvme SSD drive
**Host**	Fox HPC cluster at the University of Oslo (UiO HPC)	Google cloud	DRAGEN server V2
**Operating system**	Rocky-Linux-9	L4 and H100: Debian 11<br>A100: Rocky-Linux-9	Centos 7

- Nodes in the UiO-HPC cluster were not used in exclusive mode (the node may have shared with other users during the analysis).

- In the Google Cloud Platform (GCP), the G2 and A3 machines were created in a multi-tenant environment, meaning they shared the underlying physical hardware with VMs from other projects. However, the resources allocated to each machine, such as vCPUs and memory, are dedicated to that machine and are not shared

with others.

- The memory allocation of the CPU-only pipeline was initially determined following the configurations in https://github.com/nf-core/sarek. If a process fails due to exceeding the memory limit, it was resubmitted with an increased memory allocation.

- GCP provides L4 GPUs in G2 machines with 196 GB memory configuration. The GPUs in G2 machine were not connected via NVLink.

- The compute node of the UiO HCP cluster with four A100 GPUs offers 412 GB of resident memory. A100 GPUs were not connected via NVLink.

- The GCP provides H100 GPUs on A3 machines with eight GPUs and 1,872 GB memory configurations. GPUs in the A3 machine were connected via NVLink.

Supplementary Table S3: Runtime performance metrics

**a) Read mapping**					
**Sample**	**CPU**	**L4**	**A100**	**H100**	**DRAGEN**
NA12812	267.03	19.05	10.9	10.02	12.44
NA12778	334.2	26.02	14.98	14.15	14.08
NA12843	354.32	30.6	17.87	15.5	15.25
NA12829	507.82	45.12	26.98	24.88	21.71
NA12890	1052.92	93.9	48.27	34.78	36.23
NA12891	1128.73	103.17	60.55	39.27	39.98
NA12878	1206.48	102.78	60.52	40.22	38.85
NA12877	1266.82	108.13	53.95	43.17	41.14
NA12892	1261.37	109.77	63.58	42.95	41.99
NA12889	1285.2	123.33	60.1	45.38	43.17
**b.1) Variant calling via HC or DRAGEN GSVC**					
**Sample**	**CPU**	**L4**	**A100**	**H100**	**DRAGEN**
NA12812	556.55	5.63	4.75	4.5	7.88
NA12778	613.88	5.92	5.42	4.87	7.84
NA12843	602.7	7.05	6.43	5.67	8.19
NA12829	755.83	9.17	8.58	8	12.09
NA12890	1285.12	21.28	15.9	13.83	19.85
NA12891	1088.73	19.47	17.1	13.83	22.04
NA12878	1111.22	20.67	17.6	14	21.44
NA12877	1153.62	19.97	17.58	14.67	22.91
NA12892	1150.27	23.4	18.77	15.32	23.23
NA12889	1345.18	18.65	19.38	15.17	23.75
**b.2). Variant calling via DV**					
**Sample**	**CPU**	**L4**	**A100**	**H100**	**DRAGEN**
NA12812	252.38	9.58	4.38	4.15	NA
NA12778	337.75	12.47	5.98	6.08	NA

NA12843	290.53	11.97	5.18	6.65	NA
NA12829	313.45	17.08	7.5	9.82	NA
NA12890	428.12	26.53	13.6	14.77	NA
NA12891	465.07	28.8	17.4	22.47	NA
NA12878	435.2	30.03	16.9	14.58	NA
NA12877	429.22	29.98	15.28	16.37	NA
NA12892	465.07	31.58	17.7	16.05	NA
NA12889	488.38	33.48	17.62	20.07	NA
**c.) Total processing time: Read mapping & HC or DRAGEN GSVC**					
**Sample**	**CPU**	**L4**	**A100**	**H100**	**DRAGEN**
NA12778	948.08	117.59	20.4	19.02	21.92
NA12812	823.58	24.68	15.65	14.52	20.32
NA12829	1263.65	54.29	35.56	32.88	33.8
NA12843	957.02	37.65	24.3	21.17	23.44
NA12877	2420.44	128.1	71.53	57.84	64.05
NA12878	2317.7	123.45	78.12	54.22	60.29
NA12889	2630.38	141.98	79.48	60.55	66.92
NA12890	2338.04	115.18	64.17	48.61	56.08
NA12891	2217.46	122.64	77.65	53.1	62.02
NA12892	2411.64	133.17	82.35	58.27	65.22

Processing times (minutes) for 10 WGS samples across pipeline stages: (a) read mapping, (b.1) variant calling (HC/DRAGEN GSVC), (b.2) variant calling (DV), and (c) total time (read mapping + HC/DRAGEN GSVC). Compared pipelines: CPU-only, Parabricks (L4, A100, H100 GPUs), and DRAGEN.

Supplementary Table S4: Parabricks DV v4.1.0-1 vs v4.3.0-1 on H100 GPUs

According to the Parabricks version 4.3.1-1 documentation, DV was released with improved performance through increased GPU utilization and kernel optimization (https://docs.nvidia.com/clara/parabricks/latest/whatsnew/whatsnew4.3.1-1.html). The following table reports the runtime comparison of the version implemented in our Parabricks pipeline (4.1.0-1) and the updated version (4.3.0-1).

	Run-time (minutes)	
**Sample ID**	**Parabricks DV.v4.1.0-1**	**Parabricks DV.v4.3.0-1**
NA12878	14.58	16.05
NA12890	14.77	16.13
NA12892	16.05	17.87

Supplementary Figures

Supplementary Figure S1: Jobanalyzer evaluation via high-frequency profiling

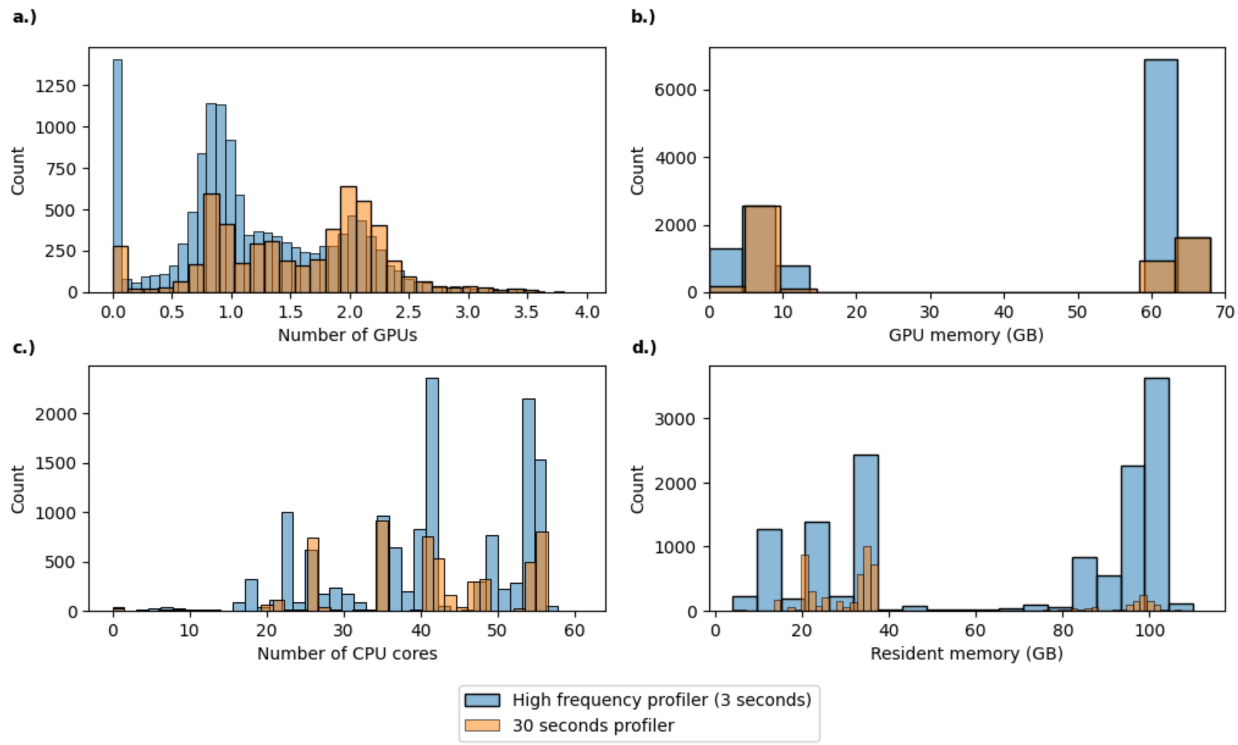

Resource usage patterns captured by high-frequency (3s intervals, blue) and low-frequency (30s intervals, orange) profiling of Parabricks on L4 GPU VMs. (a) GPU count usage, (b) GPU memory usage, (c) CPU core usage, and (d) resident memory usage. X-axises represent resource usage values divided into bins/intervals and y-axises represent the counts in each bin.

Supplementary Figure S2: Speedup vs Depth of coverage of WGS samples

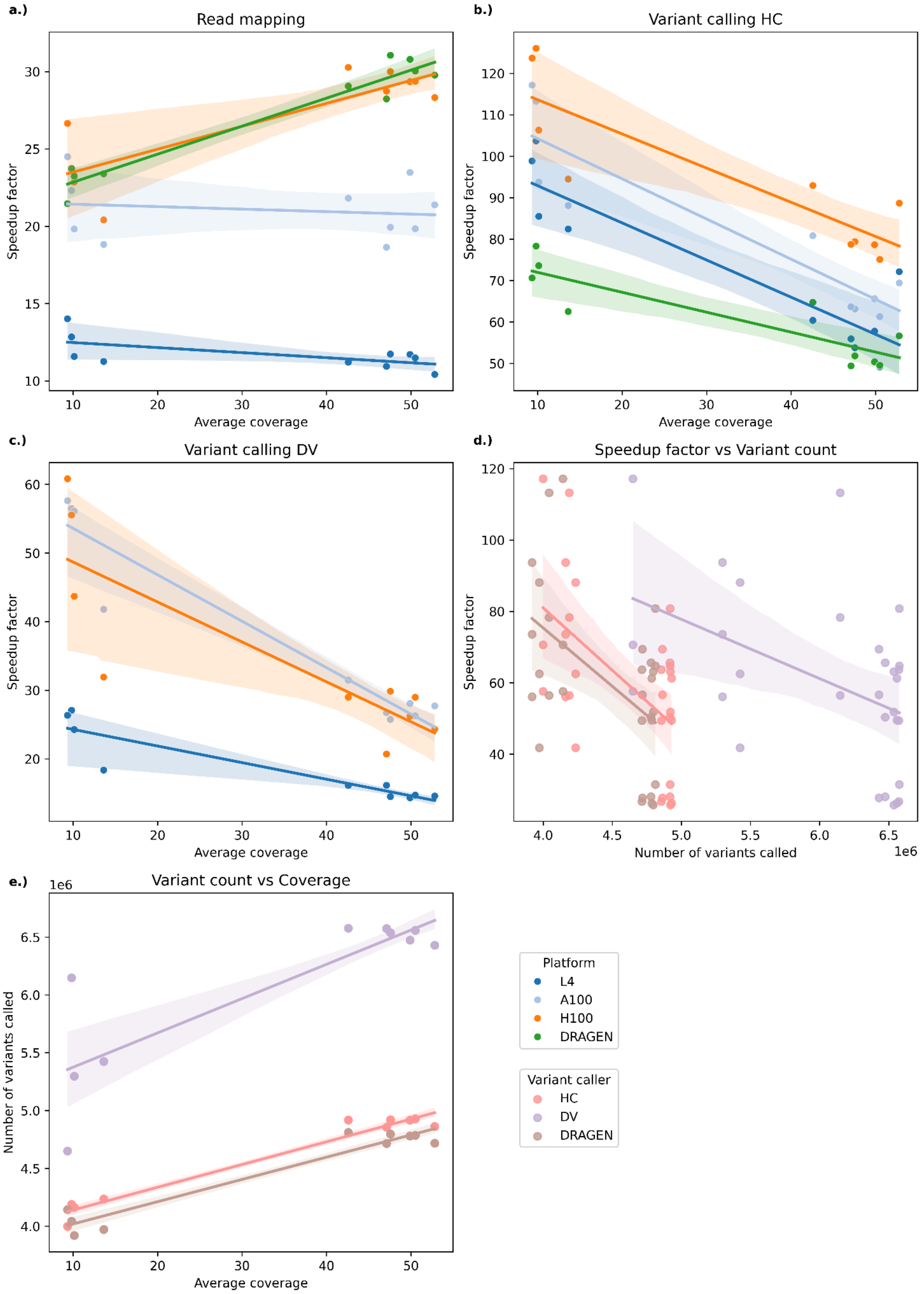

Supplementary Figure S3: GPU and GPU memory usage vs profiling time indices

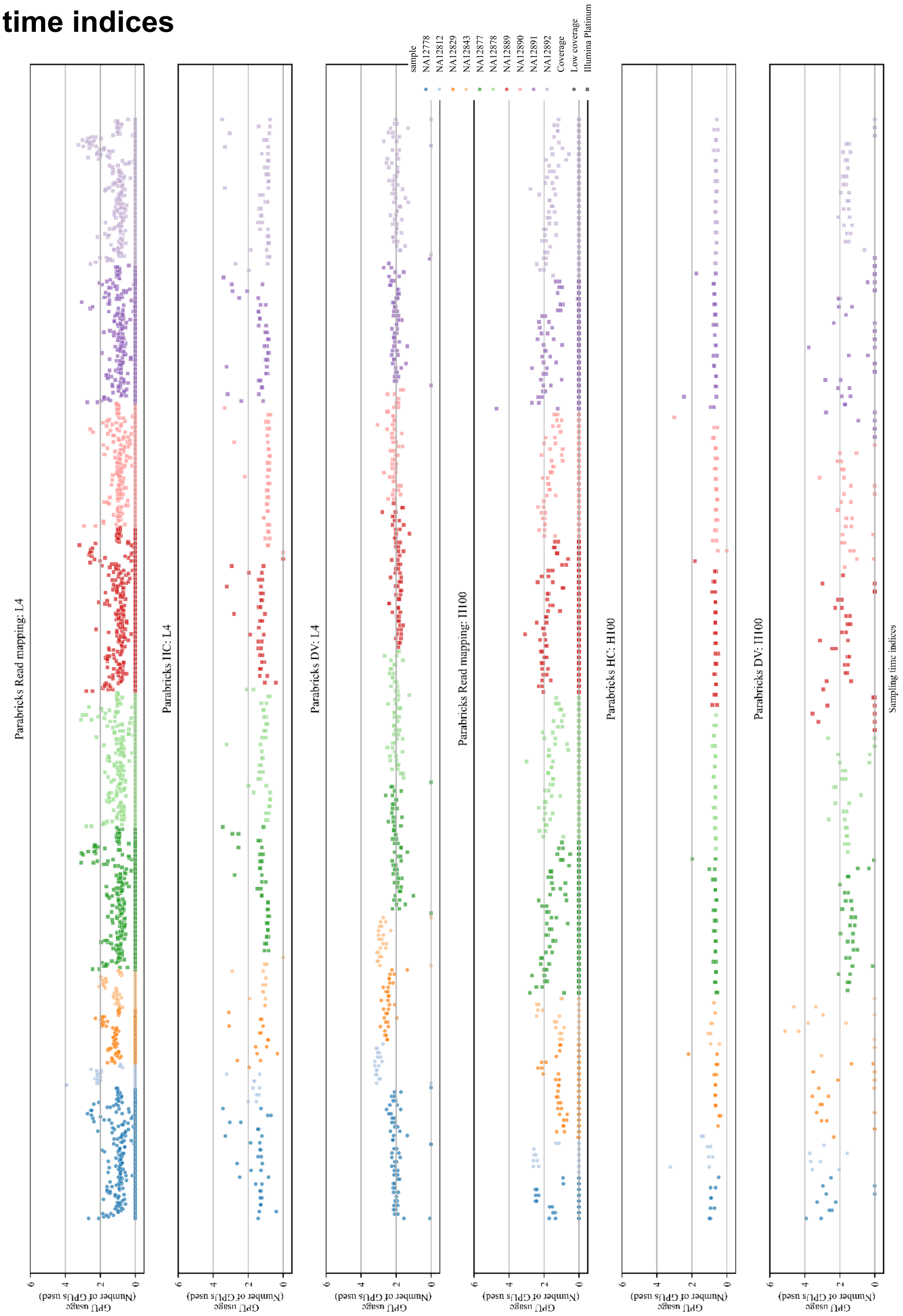

Supplementary Figure S4: Disk I/O characteristics along the profiling time indices

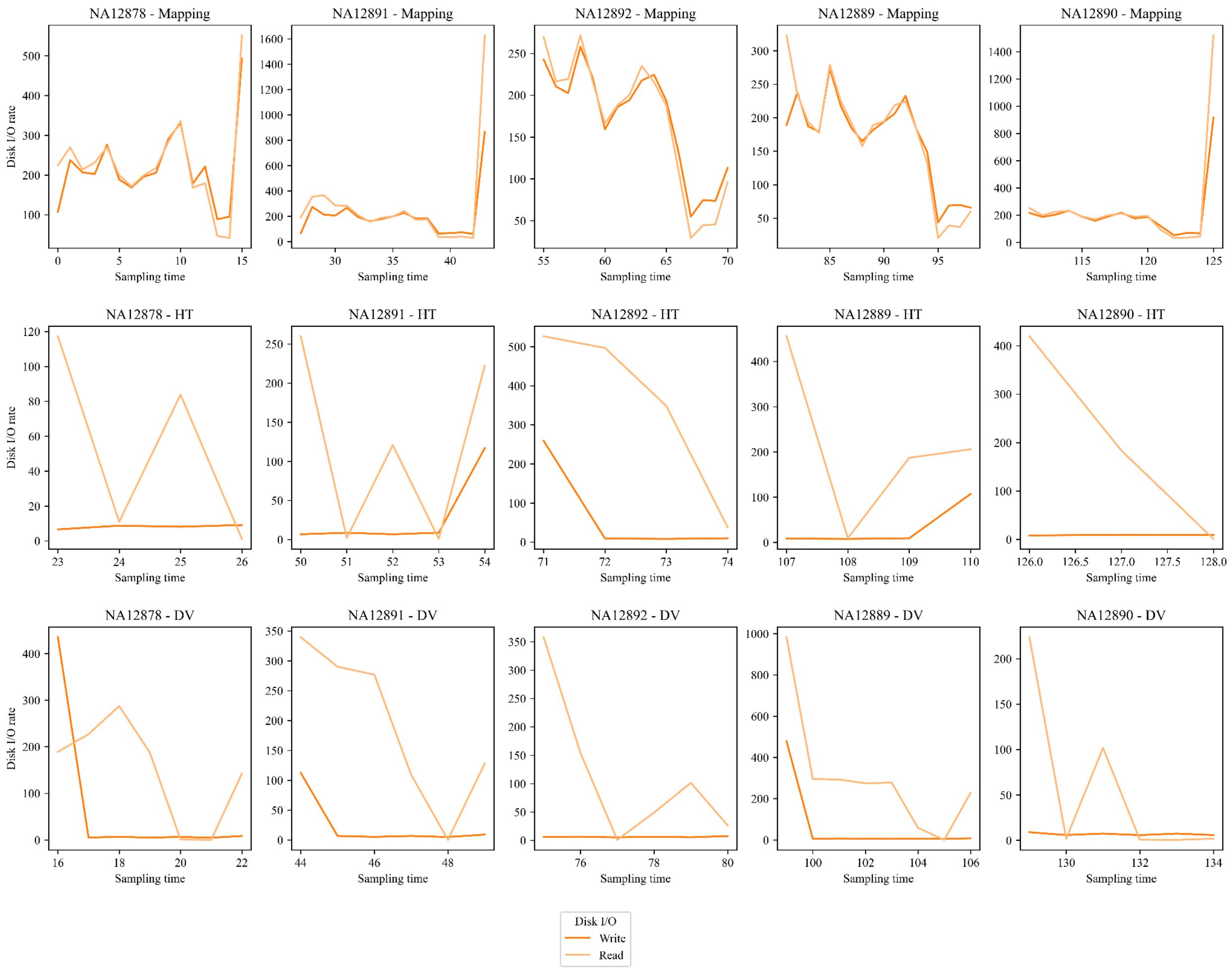

Supplementary Figure S5: Parabricks speedup on A100 and H100 GPUs relative to L4

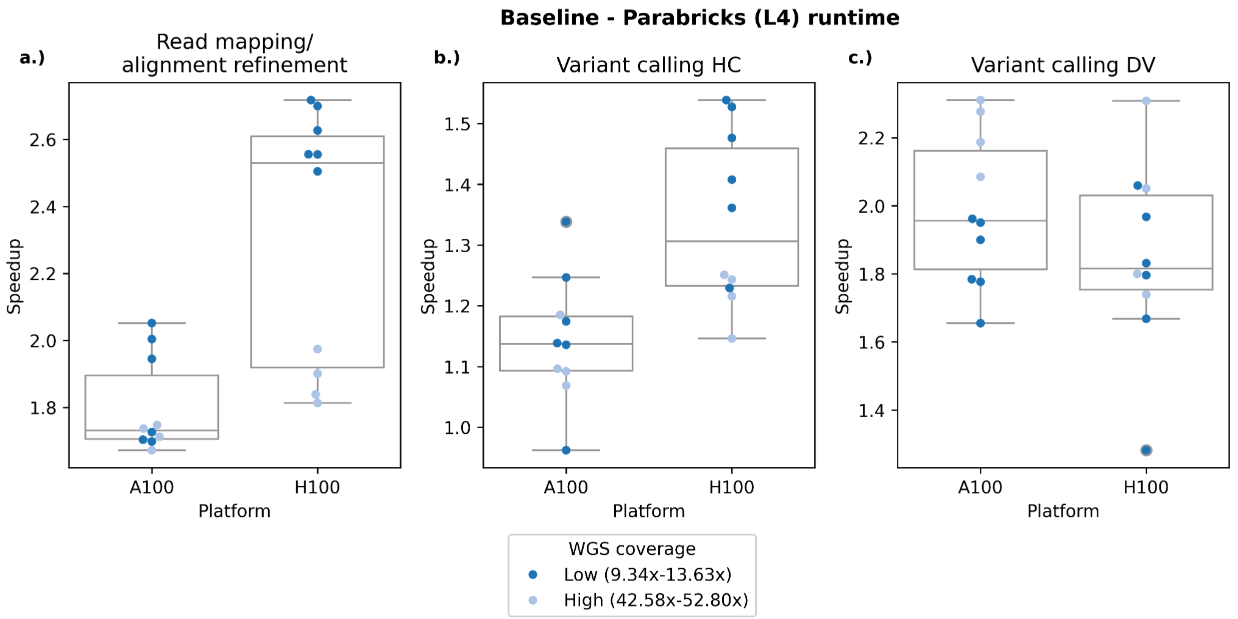

Supplementary Text

Supplementary Text 01: Parabricks original implementation vs optimized workflows

Supplementary Text 01. Figure 1: GPU usage comparison

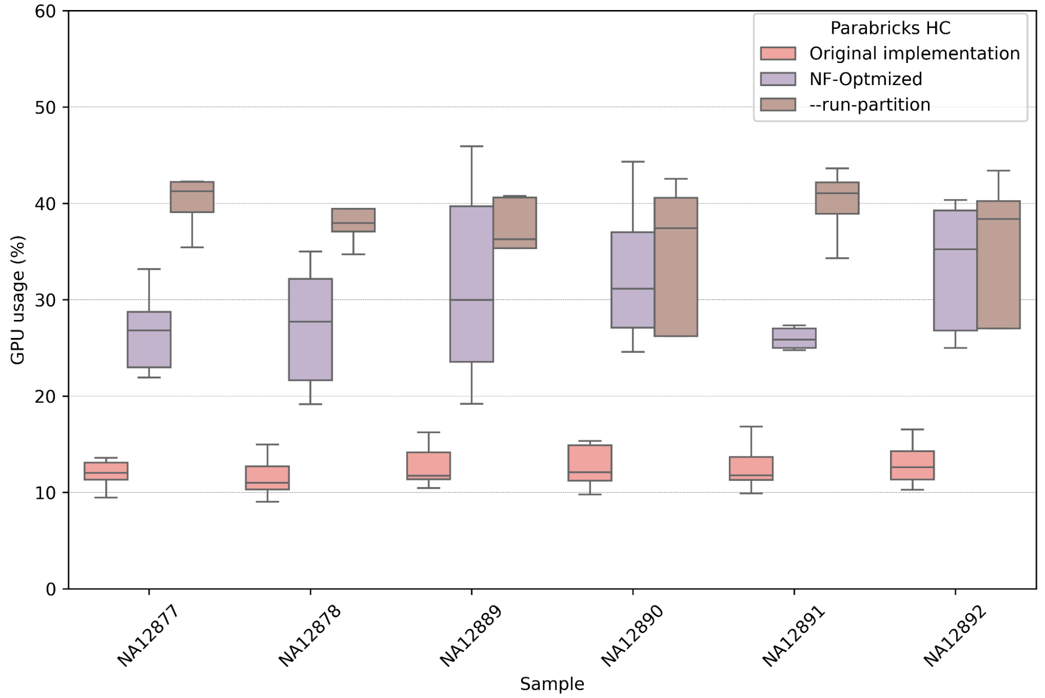

Supplementary Text 01. Figure 2: Disk I/O usage of Parabricks HC with run-partition

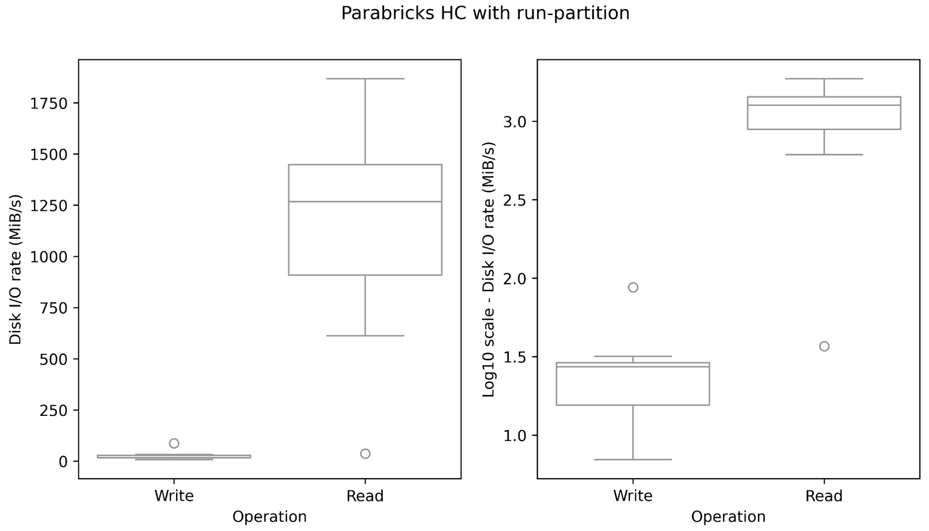

Supplementary Text 01. Table 1: Runtime performance and speedup comparison; Baseline - CPU-only pipeline

**Sample**	**CPU HC Run time (mins)**	**Parabricks HC**					
		**Run time (mins)**			**Speedup**		
		**Original**	**run-partition**	**NF-Optimized**	**Original**	**run-partition**	**NF-Optimized**
NA12890	1285.12	13.83	3.90	5.53	92.92	329.52	232.25
NA12891	1088.73	13.83	4.10	6.08	78.72	265.54	178.97
NA12878	1111.22	14	4.30	5.17	79.37	258.42	215.07
NA12877	1153.62	14.67	4.70	5.80	78.64	245.45	198.90
NA12892	1150.27	15.32	4.40	5.77	75.08	261.43	199.47
NA12889	1345.18	15.17	4.60	5.83	88.67	292.43	230.60

Supplementary Text 01. Table 2: Runtime performance and speedup comparison; Baseline - Parabricks pipeline on L4 GPUs

**Sample**	**Parabricks HC on L4 GPUs**	**Parabricks HC on H100 GPUs**			
		**run-partition**		**NF-Optimized**	
	**Runtime**	**Runtime**	**Speedup**	**Runtime**	**Speedup**
NA12890	21.28	3.90	5.46	5.53	3.85
NA12891	19.47	4.10	4.75	6.08	3.20
NA12878	20.67	4.30	4.81	5.17	4.00
NA12877	19.97	4.70	4.25	5.80	3.44
NA12892	23.40	4.40	5.32	5.77	4.06
NA12889	18.65	4.60	4.05	5.83	3.20

Supplementary Text 02: Reproducibility of Parabricks pipeline

The Parabricks pipeline was also run at the Argonne Leadership Computing Facility (ALCF) Polaris supercomputer resource for the 10 samples. Polaris is a 560 node HPE Apollo 6500 Gen 10+ based system. Each node has a single 2.8 GHz AMD EPYC Milan 7543P 32 core CPU with 512 GB of DDR4 RAM and four NVIDIA A100 (40GB) GPUs connected via NVLink.

We compared the results from the Polaris supercomputer resource with the Parabricks A100 runtimes reported on HPC cluster with AMD EPYC 7702, 64-Core Processors (UiO HPC).

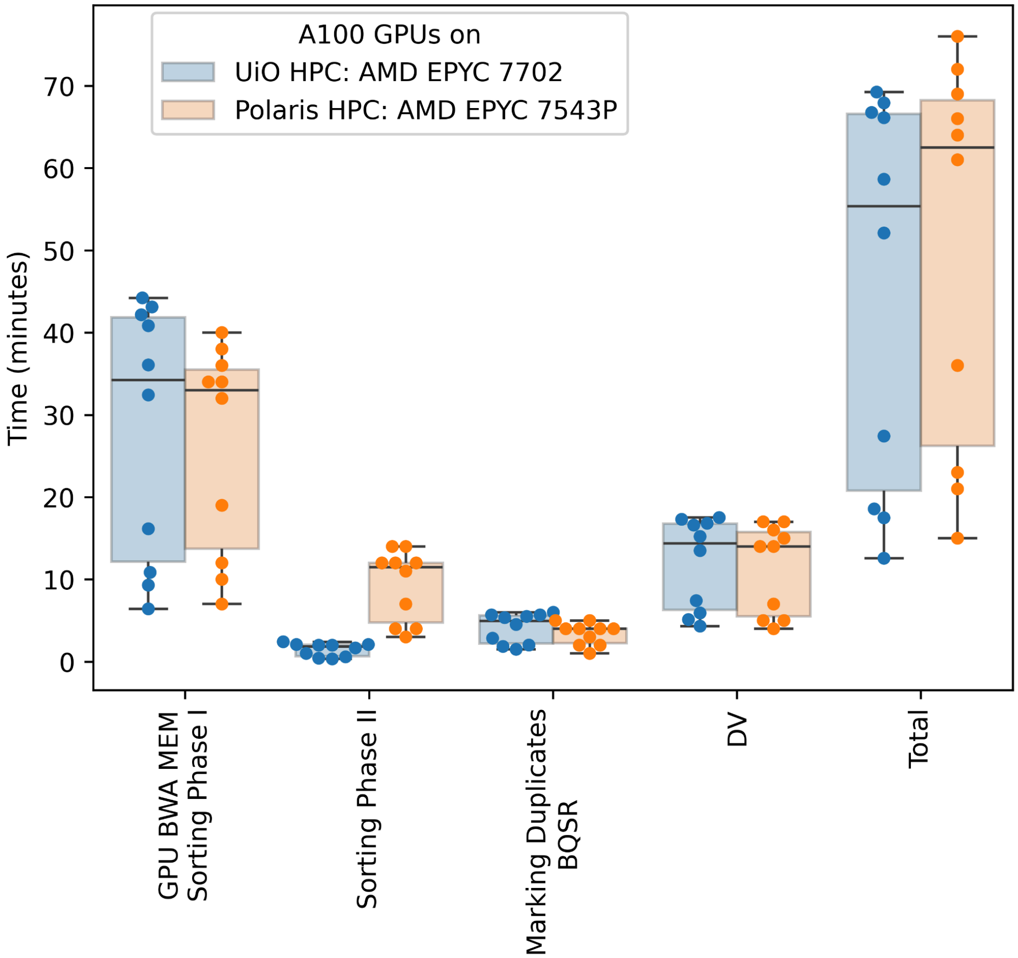

As highlighted in the figure, the two A100 GPU implementations showed similar overall runtimes. We also noticed that “Sorting Phase I” consistently had the lowest runtimes on the UiO HPC cluster. However, runtimes of all other steps in the Parabricks pipeline were shorter on Polaris HPC cluster than on UiO HPC cluster. The total runtimes of both implementations were in comparable ranges.

Supplementary Text 03: Cost comparison

Cost of running Parabricks pipeline on GCP

Estimate: GCP VM with four L4 (24GB) GPUs; 48 CPU-cores; 192 GB memory

Monthly estimate

$2,926.22

That's about $4.01 hourly

Pay for what you use: no upfront costs and per second billing

Item	Monthly estimate
48 vCPU + 192 GB memory	$1,285.90
4 NVIDIA L4	$1,635.32
50 GB balanced persistent disk	$5.00
Snapshot schedule	Cost varies
Total	$2,926.22

Estimate: GCP Spot-VM with eight (80GB) GPUs; 208 CPU-cores; 1.8TB memory

Monthly estimate

$35,604.87

That's about $48.77 hourly

Pay for what you use: no upfront costs and per second billing

Item	Monthly estimate
208 vCPU + 1,872 GB memory	$3,797.37
8 NVIDIA H100 80GB	$31,466.50
6,000 GiB Local SSD disks	$336.00
50 GB balanced persistent disk	$5.00
Snapshot schedule	Cost varies
Total	$35,604.87

- Note that the above estimates do not include costs for disks especially as they vary depending on the type and size

Cost of running DRAGEN pipeline

Estimate: DRAGEN software and hardware platform

- DRAGEN server: ~90,000 USD (with 3 years of support)
- DRAGEN - Level 1 annual license: 12 000 USD for 100,000 GigaBases
- DRAGEN - Level 3 annual license: 67 000 USD for 500,000 GigaBases

Cost of running Parabricks pipeline on HPC cluster

Estimating GPU Costs for Local Infrastructure

Determining the cost of GPU usage on a local infrastructure presents unique challenges since there is no established pricing model. While CPU hours incur charges, GPU usage does not carry additional costs. Using market prices of GPUs for estimation would be inappropriate, as the analysis was conducted on an HPC cluster where GPUs are managed by a scheduler, allocated for specific timeframes, and released to other users upon task completion.

For comparison purposes, an NVIDIA A100 GPU costs approximately 15,600 USD and typically has a 5-year lifespan. According to our funding agreement, we estimate an equivalent amount for maintenance costs:

- Total estimated cost of an NVIDIA A100 GPU including maintenance for 5 years = 31,200 USD
- Estimated hourly cost per GPU = (31,200/5)/(365*24) = 0.71 USD

As a reference point, GPU usage on the LUMI supercomputer in the academic category costs 1.53 NOK (0.13 USD) per hour (https://www.sigma2.no/user-contribution-model). Based on these figures, we can reasonably estimate that the GPU cost falls between 0.13 - 0.71 USD per hour.

Cost of analyzing 100,000 GigaBases using Parabricks pipeline on HPC cluster

The DRAGEN pricing model includes the cost for the server and the license to analyze 100,000 GigaBases (specified earlier). For the purpose of comparison, we calculated the cost of analyzing 100,000 GigaBases using Parabricks on A100 GPUs available in an HPC cluster.

Supplementary Text 03. Table 1: Calculating the cost of processing 100,000 GigaBases (GBs) using the number of bases aligned and runtime on A100 GPUs of each sample

Sample	Aligned bases (GB)	Run time on A100 (Hours)	Bases (GB) per hour	Hours per 100k GB bases	Cost (USD)	Cost (USD) per 100k GB bases
NA12778	29.86	0.34				
NA12812	27.86	0.26				
NA12829	40.31	0.59				
NA12843	30.92	0.41				
NA12877	150.54	1.19				
NA12878	145.88	1.30				
NA12889	158.91	1.32				
NA12890	130.25	1.07				
NA12891	141.83	1.29				
NA12892	155.82	1.37				
Total	1,012.18	9.15				

Bases in GB processed in 1 hour			110.58			
Hours required to process 100000 GB bases				904.33		
Cost of A100 GPU per hour					0.13 to 0.71	***117.56 to 642.08***

Estimated cost of Parabricks on A100 GPUs in HPC cluster in a pricing model comparable to DRAGEN

- NVIDIA A100 GPU server and 5 year maintenance cost = 31,200 USD
- Cost of processing 100,000 GigaBases = 642 USD

Note: As per NVIDIA Parabricks v4.4.0, licensing changes are not applicable to use NVIDIA Parabricks containers. However, the users requiring enterprise support should purchase NVIDIA AI Enterprise licenses. (https://docs.nvidia.com/clara/parabricks/4.4.0/whatsnew/licensingchanges_v4.0.0-1.html).

Supplementary Text 03. Table 2: Cost comparison summary

	Hourly rate	Cost: Local Server	Cost: Processing 100,000 GigaBases
**Google Cloud Platform compute instances**			
Four L4 (24GB) GPUs; 48 CPU-cores; 192 GB memory	4.01 USD	-	-
Spot-VM with eight (80GB) H100 GPUs; 208 CPU-cores; 1.8TB memory	48.77 USD	-	-
**Local server infrastructure**			
Parabricks on A100 GPUs in HPC cluster	-	31,200 USD *(with 5-year maintenance)*	642 USD
DRAGEN server with Level-1 annual license	-	~90,000 USD *(with 3 years of support)*	12 000 USD

Supplementary Text 04: Tools available in Parabricks vs DRAGEN

Process	Parabricks	DRAGEN
Raw data Quality Control (QC)	-	FASTQC
FASTQ/BAM Processing	fq2bam	DRAGEN mapping
	-	Read Trimming
	ApplyBQSR	-
SNV and indel Calling	haplotypecaller	GSVC
	deepvariant	-
	-	Targeted Callers
Structural variant calling	-	CNV pipeline
Quality Control (QC) matrices	BAMmetrics	QC Metrics Reporting
	CollectMultipleMetrics	
Oxford Nanopore pipeline	ont_germline	-
PacBio pipeline	pacbio_germline	-

Links to the DRAGEN tools:

- FASTQC: https://help.dragen.illumina.com/product-guides/dragen-v4.3/dragen-dna-pipeline/dragen-fastqc
- Mapping: https://help.dragen.illumina.com/product-guides/dragen-v4.3/dragen-dna-pipeline/dna-map-align
- Read Trimming: https://help.dragen.illumina.com/product-guides/dragen-v4.3/dragen-dna-pipeline/read-trimming
- GSVC: https://help.dragen.illumina.com/product-guides/dragen-v4.3/dragen-dna-pipeline/small-variant-calling
- Targeted Callers:

https://help.dragen.illumina.com/product-guides/dragen-v4.3/dragen-dna-pipeline/targeted-caller
- CNV pipeline: https://help.dragen.illumina.com/product-guides/dragen-v4.3/dragen-dna-pipeline/cnv-calling
- QC Metrics Reporting: https://help.dragen.illumina.com/product-guides/dragen-v4.3/dragen-dna-pipeline/qc-metrics-reporting

Links to Parabricks tools:

- FQ2BAM: https://docs.nvidia.com/clara/parabricks/latest/documentation/tooldocs/man_fq2bam.html#man-fq2bam
- ApplyBQSR: https://docs.nvidia.com/clara/parabricks/latest/documentation/tooldocs/man_applybqsr.html#man-applybqsr
- HaplotypeCaller: https://docs.nvidia.com/clara/parabricks/latest/Documentation/ToolDocs/man_haplotypecaller.html#man-haplotypecaller
- DeepVariant: https://docs.nvidia.com/clara/parabricks/latest/Documentation/ToolDocs/man_deepvariant.html#man-deepvariant
- BAMmetrics: https://docs.nvidia.com/clara/parabricks/latest/Documentation/ToolDocs/man_bammetrics.html#man-bammetrics
- CollectMultipleMetrics: https://docs.nvidia.com/clara/parabricks/latest/Documentation/ToolDocs/man_collectmultiplemetrics.html#man-collectmultiplemetrics
- ONT_germline: https://docs.nvidia.com/clara/parabricks/latest/documentation/tooldocs/man_ont_germline.html#
- Pacbio_germline: https://docs.nvidia.com/clara/parabricks/latest/documentation/tooldocs/man_pacbio_germline.html#man-pacbio-germline

Parabricks and DRAGEN offer comprehensive toolsets for germline NGS data processing but with distinct capabilities. In the initial processing stages, DRAGEN uniquely includes FASTQC and Read Trimming capabilities, whereas “ApplyBQSR” as per best practices was available only in Parabricks. In SNV and indel calling, Parabricks uniquely offers DeepVariant while DRAGEN provides specialised targetted callers that detect variants in regions with high sequence homology. Additionally, DRAGEN

differentiates itself from Parabricks by providing a pipeline for structural variant calling. Notably, Parabricks exclusively provides support for long-read sequencing technologies through Oxford Nanopore and PacBio pipelines.
